# Applying a brain-computer interface to support motor imagery practice in people with stroke for upper limb recovery: a feasibility study

**DOI:** 10.1186/1743-0003-7-60

**Published:** 2010-12-14

**Authors:** Girijesh Prasad, Pawel Herman, Damien Coyle, Suzanne McDonough, Jacqueline Crosbie

**Affiliations:** 1 Intelligent Systems Research Centre (ISRC), University of Ulster, Magee Campus, Derry, N. Ireland, UK; 2 Health & Rehabilitation Sciences Research Institute, University of Ulster, Jordanstown Campus, Newtonabbey, N. Ireland, UK

## Abstract

**Background:**

There is now sufficient evidence that using a rehabilitation protocol involving motor imagery (MI) practice in conjunction with physical practice (PP) of goal-directed rehabilitation tasks leads to enhanced functional recovery of paralyzed limbs among stroke sufferers. It is however difficult to confirm patient engagement during an MI in the absence of any on-line measure. Fortunately an EEG-based brain-computer interface (BCI) can provide an on-line measure of MI activity as a neurofeedback for the BCI user to help him/her focus better on the MI task. However initial performance of novice BCI users may be quite moderate and may cause frustration. This paper reports a pilot study in which a BCI system is used to provide a computer game-based neurofeedback to stroke participants during the MI part of a protocol.

**Methods:**

The participants included five chronic hemiplegic stroke sufferers. Participants received up to twelve 30-minute MI practice sessions (in conjunction with PP sessions of the same duration) on 2 days a week for 6 weeks. The BCI neurofeedback performance was evaluated based on the MI task classification accuracy (CA) rate. A set of outcome measures including action research arm test (ARAT) and grip strength (GS), was made use of in assessing the upper limb functional recovery. In addition, since stroke sufferers often experience physical tiredness, which may influence the protocol effectiveness, their fatigue and mood levels were assessed regularly.

**Results:**

Positive improvement in at least one of the outcome measures was observed in all the participants, while improvements approached a minimal clinically important difference (MCID) for the ARAT. The on-line CA of MI induced sensorimotor rhythm (SMR) modulation patterns in the form of lateralized event-related desynchronization (ERD) and event-related synchronization (ERS) effects, for novice participants was in a moderate range of 60-75% within the limited 12 training sessions. The ERD/ERS change from the first to the last session was statistically significant for only two participants.

**Conclusions:**

Overall the crucial observation is that the moderate BCI classification performance did not impede the positive rehabilitation trends as quantified with the rehabilitation outcome measures adopted in this study. Therefore it can be concluded that the BCI supported MI is a feasible intervention as part of a post-stroke rehabilitation protocol combining both PP and MI practice of rehabilitation tasks. Although these findings are promising, the scope of the final conclusions is limited by the small sample size and the lack of a control group.

## Background

Over 20 M people suffer from stroke annually world-wide and up to 9 M stroke survivors may suffer from permanent upper limb paralysis, which may significantly impact their quality of life and employability [1]. There is now sufficient evidence that that physical practice (PP) (i.e. real movement) along with motor imagery (MI) practice (often called mental practice) of a range of therapeutic (or motor) tasks can lead to improvements in reaching, wrist movements and isolated movements of the hands and fingers and object manipulation of the impaired upper limb [2-4] and although this evidence is promising it is still limited in many respects [5]. One of the challenges of using MI practice is confirming patient engagement on-line so as to help him/her undertake MI with sufficient focus. A direct non-invasive approach to confirming MI is to assess the modulation of brainwaves obtained from the continuous measurement of electroencephalography (EEG) signals during the MI practice as part of a brain-computer interface (BCI). Although EEG-based BCI approach devised based on the detection of EEG correlates of MI (measured as MI task classification accuracy (CA)) has been widely investigated in healthy subjects [6,7], it is yet to be systematically explored in stroke sufferers. Also, it has been found that a substantially large proportion of subjects may not be very good at performing MI, resulting in a moderate CA obtained with an MI-based BCI system in initial few sessions [8]. But, through practice over several sessions, most subjects may significantly improve their performance [9]. It is however not known how this initial moderate level of performance affects rehabilitation outcomes, especially if the subjects perform MI tasks with the support of neurofeedback from a BCI with moderate CA. A moderate accuracy feedback may frustrate the subject and thus cause more of a distraction rather than assistance in performing MI of rehabilitative tasks. There is also a concern that with an inaccurate feedback the subject may be executing MI practices that affect an unintended brain hemisphere and thus hinder the recovery process.

Very few EEG-based BCI studies report involvement of stroke sufferers [10-13]. A small set of preliminary results in [11] demonstrates that a single-trial analysis represents an appropriate method to detect task-related EEG patterns in stroke patients. It is also reported that during physical motor execution as well as MI, mainly the frequency components lower β (16-22 Hz) and μ (9-14 Hz) play an important role for an intact as well as a paretic hand. In [10], an EEG BCI supported functional electrical stimulation (FES) platform is reported with the aim of training upper limb functions of a chronic stroke sufferer. In this study, two chronic patients participated attaining an error rate of BCI control less than 20%. However, no evidence is reported that the BCI use resulted in any gain in upper limb recovery. The use of magnetoencephalography (MEG) based BCI by patients with chronic stroke for controlling a hand orthosis attached to the paralysed hand is reported by Buch et al. [14]. In this study, the MI induced modulations in 10-15 Hz sensorimotor rhythms (SMRs) were quantified to serve as features for devising the BCI. Patients received visual and kinaesthetic feedback of their brain activity. 90% of the patients were able to voluntarily control the orthosis in 70-90% of the trials after 20 hours of training. In the course of training the ipsilesional brain activity increased, and spasticity decreased significantly. However, hand movement without the orthosis did not improve, i.e. no functional recovery was observed. In [12,13], a controlled trial was reported involving 12 stroke patients undertaking a robot supported upper extremity exercises over a period of 20 weeks. A BCI driven switch was used to switch on the exercise sessions. No significantly higher increase in rehabilitation outcome measures was achieved with the BCI supported protocol when compared to that using robots alone. Thus no BCI supported study consisted of a rehabilitation protocol involving a combination of PP and MI practice. Mostly, an MI BCI has been used as a switch to initiate the rehabilitation exercise and then the actual exercise involving motor execution is performed with an external robotic support.

The research question (or hypothesis) for the study presented in this paper was whether it is feasible to make use of an EEG-based BCI generated neurofeedback to support patient's engagement during an MI practice performed as part of a post-stroke rehabilitation protocol combining both PP and MI practice. To this end, the study was aimed at determining recruitment adherence and drop-out issues; integrating an EEG-based BCI with the MI-based rehabilitation protocol; piloting of the methodological and intervention procedures; assessing qualitative effects of the intervention on participants; and identifying most appropriate motor outcomes for monitoring incremental motor recovery. As there was no prior knowledge available about the interventions to be used, it was thought vital in the initial stage to place major emphasis on testing the acceptability and adherence with the intervention before planning a large-scale controlled trial.

## Methods

### Selection of Participants

The aim of the study was to work towards devising a rehabilitation protocol that helps in functional recovery of upper limb paralysis of stroke sufferers whose motor cortex has stopped reorganizing. As an auto-recovery is normally not expected beyond the first year, any individuals with some degree of upper extremity motor impairment and who had sustained a stroke at least a year before, were considered for inclusion onto the study. Potential participants were excluded if they were medically unstable at the time of assessment; had any history of epilepsy; were unable to follow a two-step command; showed any signs of confusion or neglect (evidenced by a Hodgkinson mini-mental test score (HMMS)) [15] of less than 7/10 and Star cancellation test (Star CT) score [16] of less than 48/52 respectively (Table 1). Ethical approval for the study was gained through the University of Ulster Research Ethics committee, N Ireland.

**Table 1:** Subject Baseline Demographics

*Participants*	*Age (y)*	*Gender*	*Impaired side*	*Dominant side*	*Time since stroke (m)*	*HMMS*	*STAR CT*
P1 (091153)	55	M	L	R	48	10/10	52/52

P2 (230361)	47	F	L	R	41	10/10	52/52

P3 (210151)	57	M	L	R	15	8/10	52/52

P4 (250345)	63	M	R	R	20	10/10	52/52

P5 (231237)	71	M	R	R	16	10/10	52/52

MEAN (± SD)	58.6 (8.98)				28(15.4)		

### Experimental Procedure

The experimental protocol involved a therapeutic regimen consisting of a treatment session that included both PP and MI practice of a therapeutic task. The task was decided in consultation with the participants, although most performed or imagined hand clenching. The session content was based on that described by Weiss et al. [17]. Before the beginning of each session, a trained researcher explained the task by using simple instructions and showing a video of the sequence of movements that should be performed with his/her own hands. The MI consisted of imagining the performance of motor sequences and kinaesthetic sensations associated with it while holding the upper limbs still.

On reviewing the literature regarding the length of therapy to stroke patients, it was observed that somewhat similar virtual reality (VR) mediated therapies were most commonly administered three times per week for 1-1.5 hours over a 2-4 weeks period [18]. Taking into account the logistics involved in participants travels, laboratory preparations, and data processing and analysis, it was decided to conduct 2 treatment sessions each week for a total of 6 weeks. In each treatment session, the participants first performed a sequence of PP and then MI of the same. The participant started with 10 repetitions (or trials) with the unimpaired (or less affected) upper limb followed by 10 repetitions with the impaired (or more affected) limb for both PP and MI parts of the session. This sequence was repeated with both the PP and the MI parts of a session divided into 4 runs of 40 trials. Throughout the MI session, the participants sat relaxed on their chair with their eyes open. From the second or third session onwards, the participants were provided with neurofeedback through the EEG-based BCI during the MI part of the session only. The neurofeedback was provided as part of a computer game called "ball-basket" (explained later) in which a ball falling at a constant speed from the top of the screen to the bottom within a predefined interval of 4 s during the time period of 3 s to 7 s of a trial, was required to be placed in a green target basket appearing on either the left or the right side at the bottom of a user window with the help of the MI of the respective limb. The feedback showed the direction of the ball movement as a result of the patient's MI in response to the target basket appearance. The participants were advised to keep focusing on their left or right arm/hand MI tasks, so as to manoeuvre the ball towards the green basket, while constantly maintaining the balls on the same side. The total length of the trial varies between 8 and 10 s. As a result, there is a random gap of 1 to 3 s during which the screen remains blank and participants are asked to relax.

### Design of the EEG-based BCI and Neurofeedback

**Figure 1:**
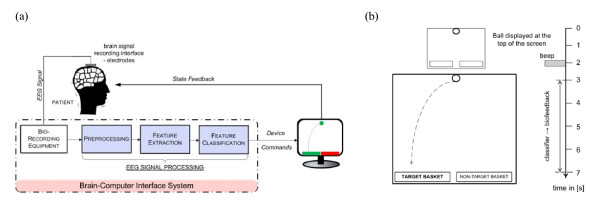
**An illustration of a Brain-Computer Interface**: **(a) **Main components of a BCI. **(b) **Timings of a ball-basket game paradigm.

A block-diagram representation of the EEG-based BCI system is shown in the Figure 1a. The BCI was designed using the data recorded from two bipolar EEG channels around C3 and C4 locations (two electrodes placed 2.5 cm anterior and posterior to C3/C4) based on the 10/20 international system. The EEG was recorded with a g.BSamp amplifier system from g.tec, Graz, Austria. In addition, an EEG cap with Ag/AgCl electrode assembly from Easycap™was utilized. EMG signals from biceps were also recorded to monitor whether there were any actual physical movements during the MI practice. MATLAB Simulink based BCI software developed in-house was employed in devising various stages of the BCI and neurofeedback system. In the preprocessing stage, the EEG signal was band-pass filtered between 0.5 and 30 Hz with the 50 Hz notch. The bio-signals were sampled at 500 Hz. The BCI closed-loop was realized through the neurofeedback provided in a computer game-like environment using the ball-basket game (Figure 1b). As shown in Figure 1b, red (non-target) and green (target) rectangles (or baskets) were displayed at the bottom of the user window at the beginning of each trial interval. After 2 s from the beginning of a trial, a ball appeared on the top of the user window and a beep sound informed the user to start attempting to manoeuvre the ball by means of his/her left/right arm/hand MI corresponding to the horizontal location of the green target basket (i.e. left vs. right). The game's objective is to place the ball in the target basket (green rectangle). During the trial period, the scalp EEG data is continuously recorded.

It is known that when the sensorimotor area of the brain is activated during the imagination of upper limb movement, there often occurs contralateral attenuation of the μ (8-12 Hz) rhythm and ipsilateral enhancement of the central β (18-25 Hz) oscillations [6,19,20]. These processes occur due to the neurophysiological mechanisms of the so-called event-related desynchronization (ERD) and event-related synchronization (ERS) [6,7,19]. The exact EEG manifestations and frequency bands of ERS and ERD may vary from subject to subject. Subject specific ERD and ERS patterns, i.e. estimates of the spectral power of C3 and C4 signals within the adjusted μ and β bands, providing best separability between left and right hand movement imaginations, were therefore acquired in this work from the recorded trials in the feature extraction stage. To this end, power spectral density (PSD) was parametrically estimated from the frequency response of the autoregressive model (of arbitrary order *n*), which was fitted to the EEG signal by solving Yule-Walker equations [21]. These linear equations relate the parameters of the autoregressive model, *a*_1_..*a_n_*, with the autocorrelation sequence γ(*k*) (*k *is the time lag).

$$\gamma \left(k\right)=\alpha_{1}\gamma \left(-k+1\right)+\ldots +\alpha_{n}\gamma \left(-k+n\right),\;k=1,..,n$$

The model parameters were found using Levinson-Durbin recursion by minimising the forward prediction error in the least-square sense. The feature separability was quantified off-line using the cross-validation estimate of the CA obtained with a linear discriminant analysis approach.

#### Designing the Feature Classifier

The EEG features extracted from the 1 s long sliding window were exploited as inputs to a two-class fuzzy logic system classifier [22] in the feature translation stage that infers the class of the associated MI. The classifier output, updated every data sample, was then directly used as the feedback signal in the ball-basket game allowing for controlling the amplitude of the horizontal component of the ball's movement (the amplitude was proportional to the classifier's output signal). The vertical component of the movement was kept at a constant value so that the ball could steadily cover the distance from the top to the bottom of the user window within a predefined interval of 4 s (i.e. from 3 s to 7 s).

The classifier was designed off-line on the EEG features extracted from the data set recorded in the previous on-line sessions. A type-2 fuzzy logic classifier was adopted in this study [23]. Analogously to classical type-1 fuzzy systems, it is defined in terms of a fuzzy rule-base and an inference mechanism that allows for processing fuzzy information to eventually generate the system output. However, unlike in conventional fuzzy models, rules are represented as type-2 fuzzy relations with extended (interval type-2) fuzzy sets [24], which provides scope for more robust handling of the variability (predominantly, long- and short-term non-stationarity) of the EEG signal dynamics. A template of a Mamdani type-2 fuzzy rule exploited in this work is the following [23]:

$$\text{IF }X_{1}\;\text{is}\;{\tilde{A}}_{1}\;\text{AND}\ldots \text{AND}\;X_{n}\;\text{is}\;{\tilde{A}}_{n}\;\text{THEN}\;class\;\text{is}\;C.$$

**Figure 2:**
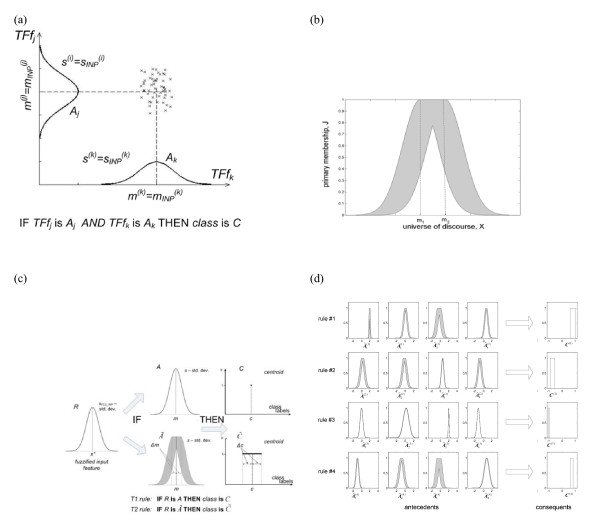
**A Type-2 Fuzzy Classifier**: **(a) **A two-dimensional cluster in the feature space and the corresponding T1 fuzzy rule. **(b) **Footprint of a Gaussian interval type-2 fuzzy set with uncertain mean *m*∈[*m_1_,m_2_*]**. (c) **Illustrative comparison of a one-rule T2FLS and T1FLS-based classifiers (Δm and Δc define the initial bounds of uncertainty modeled in the system. **(d) **Structure of a sample T2 fuzzy rule base (the domain of the antecedents' membership functions is normalised).

Fuzzy sets *X_i _*(*i *= 1,..,*n*) are conventionally fuzzified components (Gaussian type-1 fuzzy sets) of an input feature vector ***x***(spectral correlates of the ERD/ERS extracted from the μ/β bands of C3/C4 EEG channels). ${\tilde{A}}_{i}$s denote type-2 fuzzy sets and *C *is the centroid of the consequent type-2 fuzzy set representing the class that the input feature vector is assigned to. In interval type-2 fuzzy systems, the outcome is represented in terms of intervals (cf. Figure 2b). In consequence, the system has more degrees of freedom in the description of its fuzzy sets.

Fuzzy sets are determined in the fuzzy classifier's design process. Initially, clustering is performed on the extracted EEG spectral power features (in μ and β bands) using the mapping-constrained agglomerative clustering. Next, prototype classical type-1 fuzzy rules were intialised based on clustering outcome. In particular, each cluster served as a prototype for one Mamdani-type fuzzy rule. Each premise was constructed using Gaussian membership functions with the centres and widths corresponding to the cluster mean and its estimated spread, respectively, projected on the data axes. The crisp consequent was randomised between -1 and 1 (the interval borders denoting left and right MI classes, respectively). Rather small sized systems (4-8 rules) were preferred to minimize over-fitting effects and satisfy real-time computational constraints in the recall phase [22]. For the purpose of easy visualization, an example of the projection of a two-dimensional cluster of data belonging to class C on the axes corresponding to respective feature vector components (*TFf_i _*, for the two-dimensional example *i*={1,2}) and the resulting type-1 fuzzy rule (with Gaussian fuzzy sets *A_i _*defined by the means *m*(*i*)=*m_INP_*(*i*) and standard deviations *s*(*i*)=*s_INP_*(*i*) in the rule antecedent) are shown in Figure 2a. In the next step, type-1 fuzzy rules are transformed into their type-2 counterparts by substituting type-1 fuzzy sets by Gaussian interval type-2 sets (here, with uncertain mean). In particular, the so-called footprint of each interval type-2 fuzzy set (cf. Figure 2b) was obtained by applying the following set of extension formulae:

$$m_{1}\text{=}\;m_{INP}\;\text{-}\;\Delta m\text{;}\;\;\;m_{2}\;\text{=}\;m_{INP}\;\text{+}\;\Delta m;\;\;\;c_{left}=m_{OUT}–\Delta c;\;\;\;c_{right}=m_{OUT}+\Delta c;$$

where, *m_INP _*defines the centre of each corresponding Gaussian type-1 fuzzy set in the premise, and *m_OUT _*serves as the crisp output of the corresponding fuzzy rule.

The process of deriving and initialising type-2 fuzzy classifier is illustrated in Figure 2c, which compares only one-rule systems with single antecedent. As can be seen, type-1 fuzzy set *A *is replaced with type-2 fuzzy set $\tilde{A}$ Analogously, the crisp *C *centroid of type-1 rule consequent is transformed into the interval centroid $\tilde{C}$. In the final stage of designing a type-2 rule-based system, which amounts to positioning and adjusting the spread of Gaussian interval type-2 fuzzy sets in the antecedents, and adjusting the consequents' interval centroids, a gradient-based learning algorithm was employed with the mean-square error criterion. Hence, the initialised fuzzy sets were fine tuned to optimise the system's classification performance. The example type-2 rule base is shown in Figure 2d in the form of footprints of the antecedent fuzzy sets and centroids of the corresponding consequents. The detailed description of the algorithm and the structure of the type-2 fuzzy classifier can be found in [23]. For a thorough discussion of type-2 fuzzy sets and systems it is recommended to refer to [24].

### Quantification of SMR modulation effects during BCI-supported MI practice

The EEG data and the classifier's output recorded over multiple sessions were also analyzed off-line to investigate neurophysiological effects of BCI-supported MI practice and identify their correlations with outcome measures. In particular, the ERD and ERS phenomena associated with MI were main target. To this end, the spectral content of EEG trials recorded over both contralateral and ipsilateral hemispheres (w.r.t. the MI) before the cue onset (reference period) and during the MI task was analyzed in each session including the first one without feedback. Trials involving artefacts, especially eye blinks in the reference interval, were excluded. Spectral analysis was performed using the Yule-Walker PSD approach within the adjusted μ and β frequency bands (following a similar method as used in the on-line computation). These adjustments were carried out to maximize the dynamic range of within-trial power fluctuations corresponding to SMR modulations. The resultant reactive frequency bands were in a strong agreement with the outcome of analogous optimization from the perspective of BCI performance.

The ERD/ERS is defined here as the ratio of signal's energy within a specified frequency band *f *(μ or β) measured during the MI task ($E_{MI}^{\left(f\right)}$) and that during the reference period ($E_{ref}^{\left(f\right)}$) [9]:

$$\text{ERD}/{\text{ERS}}_{f}=\frac{E_{MI}^{\left(f\right)}}{E_{ref}^{\left(f\right)}}.$$

ERD occurs, if the ratio is less than 1, otherwise if it is greater than 1, the phenomenon is referred to as ERS. ERD/ERS is usually evaluated as a function of time using a sliding window over the trial duration. Similar approach was adopted in this work with the window length of 2 s keeping the reference period from 0.5 s to 2.5 s. For estimating the overall effects, ERD/ERS was evaluated first for each trial and then averaged within a session (separately for left and right hand MI trials). The resultant time courses of the averaged ERD/ERS were then quantified for μ and β bands separately.

### Rehabilitation Outcome Measures

For this feasibility study we measured the following outcomes: Rate of attendance (%); Upper limb movement and motor control: Motricity Index (McI) [25], Action Research Arm Test (ARAT) [26], Nine Hole Peg Test (NHPT) [27] and Grip Strength (GS) [28]; Fatigue and mood [29]; and Qualitative Feedback. All outcomes were recorded by the same independent researcher who was trained in their use prior to the commencement of the study. Unless stated otherwise, outcomes were recorded at baseline (i.e. time-point 1 falling in the week before the intervention began (W0)), at six separate time points along with the 2^nd ^treatment session every week during the six week intervention period (W1 to W6), and at the follow up assessment approximately one week later (i.e. time-point 8 falling in the week following the intervention period (W7)).

#### Upper limb movement and motor control

The upper extremity section of McI was used in order to assess motor impairments. The test consists of a series of movement tasks completed in the sitting position. The tests are graded on a scale of 1-100. In a similar manner to the Medical Research Council scale for muscle strength, the test involves grading strength depending on the individual's ability to activate a muscle group, by moving the relevant limb through its available joint range of motion while resisting a force applied by the examiner [25].

ARAT, first described by Lyle and co-authors [26] is a commonly used measure to assess upper-extremity functional limitations in individuals with cerebral cortical injury. The following apparatus is required in order to administer the test: a chair and table, woodblocks, a cricket ball, a sharpening stone, two different sizes of alloy tubes, a washer and bolt, two glasses, a marble and a 6 mm ball-bearing. The ARAT uses an ordinal scale including 19 separate items or movement tasks. Each task is graded with 0 indicating no movement and 3 for full or normal movement. These 19 items are grouped into gross motor (9 points), grasp (18 points), grip (12 points) and pinch (18 points) tasks, with a maximum score of 57 points. A minimal clinically important difference (MCID) for ARAT has been set as 5.7 points [30].

NHPT was used to assess fine manual dexterity [27]. The apparatus required for the test includes nine pegs (7 mm diameter, 32 mm length) and a wooden board with nine holes slightly larger than the pegs placed 32 mm apart. Participants were instructed to pick up one peg at a time with the affected arm and place them into the holes as quickly as possible. The time taken for the participant to place the nine wooden dowels into nine holes on a board and to then remove them was recorded in seconds. A maximum test time of 120 seconds was allowed for each test. When a participant was unable to complete the test in this time, the number of dowels placed and removed was recorded instead. To allow for the different recording methods a six point scale was constructed for the purposes of the study (Table 2). However, an MCID has not been established for the NHPT.

**Table 2:** Ordinal 6 Point Grading Scale for the Nine Hole Peg Test.

NHPT OUTCOME	SCORE
0-30 seconds to complete	6

31-60 " "	5

61-120 " "	4

7-9 pegs in 2 min	3

4-6 pegs " "	2

1-3 pegs " "	1

0 pegs and/or void test	0

Dynamometry is accepted as a simple and reliable method for measuring muscle strength deficits after stroke. While GS is used to directly describe strength of the hand, it may also indicate the level of overall upper extremity strength [28]. Here the Baseline dynamometer (White Plains, New York 10602) was used with one measurement recorded at each time point to limit the effects of fatigue. Comparisons of handgrip strength measurements with upper limb functional tests suggest that failure to recover measurable grip strength before twenty four days is associated with the absence of useful arm function at three months [31].

#### Fatigue and Mood

Among stroke sufferers, fatigue is frequent and often severe even late after stroke [29]. In this study, fatigue was considered in a limited sense that the participants may get tired and loose attention during the session. Undergoing the therapy sessions may make the feeling of tiredness much worse. To monitor the influence of fatigue on the effectiveness of the therapy, the feeling of fatigue was assessed. It involved completing a 10 cm Visual Analogue Scale (VAS) [29,32]. The scale was marked as "No fatigue" at one end and 'Worst fatigue imaginable' at the other. As fatigue and mood are often correlated it was decided to asses each participant's mood during the intervention period. The mood was also monitored by completing a 10 cm VAS. For mood, the scale was marked as "No depression" at one end and 'As bad as I could feel', at the other. The VAS scales were recorded twice in the week before the intervention, twice per week during the intervention period and once in the follow-up week, resulting in 15 time-points.

### Scope of Data Analysis

Since this was a feasibility study involving a small number of subjects with no control group for a limited period of time, significance tests on the data could not be performed for any of the rehabilitation outcome measures. Treatment effects were assessed on a case by case basis and group mean outcome scores were computed. Adherence levels and any difficulties experienced by the participants or research staff were reported. This may be used to modify the interventions in a larger future trial.

For each participant however, EEG data was recorded over up to 12 treatment sessions and each session consisted of 160 trials having MI related EEG data of 4 s sampled at 500 Hz. Such a large data set facilitated carrying out subject-wise significant test to find whether there was statistically significant difference between ERD/ERS occurrences in the first and the last session. It also facilitated undertaking following correlation analyses.

• ERD/ERS vs CA for both left and right hand MI separately

• ERD/ERS vs rehabilitation outcomes measures.

## Results

### Participants

26 participants were screened for eligibility for this study, of this number, five met the inclusion criteria and their demographics are displayed in Table 1. The main reasons for exclusion from this study were length of time since stroke greater than 5 years, and co-existing cognitive impairment. The mean age of included participants was 59 years, with four males and one female. Three had experienced a right sided stroke (i.e. left hand side impairment), two left sided, and all were right hand dominant. The time since stroke was variable, ranging from 15-48 months, all showed good cognitive function and no perceptual difficulties.

### Adherence

The attendance rate was surprisingly high for this small group of participants given the time consuming nature of the intervention, which took on average 2 hours per session. From a patient's perspective adherence was very high, however due to technical problems with the recording equipment, it was necessary to cancel some of the sessions so the overall level of attendance was 100% for four individuals, and 92% (11/12) for one participant.

### BCI Neurofeedback Performance

**Figure 3:**
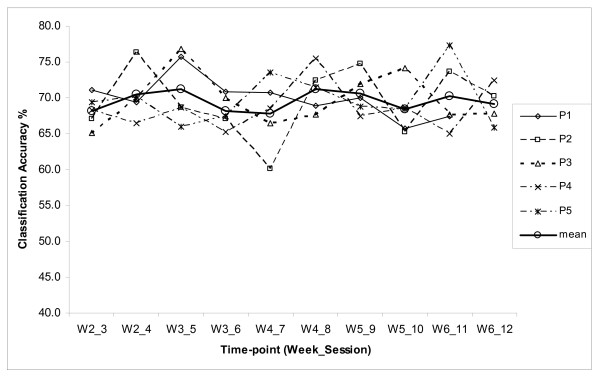
**BCI Classification accuracies over the feedback sessions**.

The neurofeedback was provided to the study participants in real-time using the aforementioned fuzzy rule-based BCI classifier. The BCI performance was evaluated based on the MI task classification accuracy (CA) rates obtained during on-line system use. The maximum CAs reported in separate runs were averaged within each session (four 40-trial runs) for every participant. These CA values are plotted in Figure 3. The stroke participants were novice BCI users. The session CA values are in the range 60-75%. This moderate CA range obtained with stroke patients is commonly observed in novice BCI users. In a previous study, using a similar BCI system design with the same ball-basket feedback paradigm, trials were also conducted on six healthy novice participants over ten sessions. These participants achieved a CA range of 69.2 ± 4.6% [22], which is very similar to that of stroke patients. It is also to be noted that a similar CA variation range was also observed in [14] in the first 10 sessions, where 8 stroke sufferers participated in an MEG based BCI study. With regard to the course of the CA statistics over experimental sessions, some fluctuations were observed for every participant. This tendency is characteristic of early stages of learning how to control BCI by novice users. The effect of learning gain on the CA performance due to undertaking MI practices for up to 12 sessions is however insignificant. It should also be noted that no follow-up evaluation was conducted to examine whether this trend corresponds with other outcome measures.

**Figure 4:**
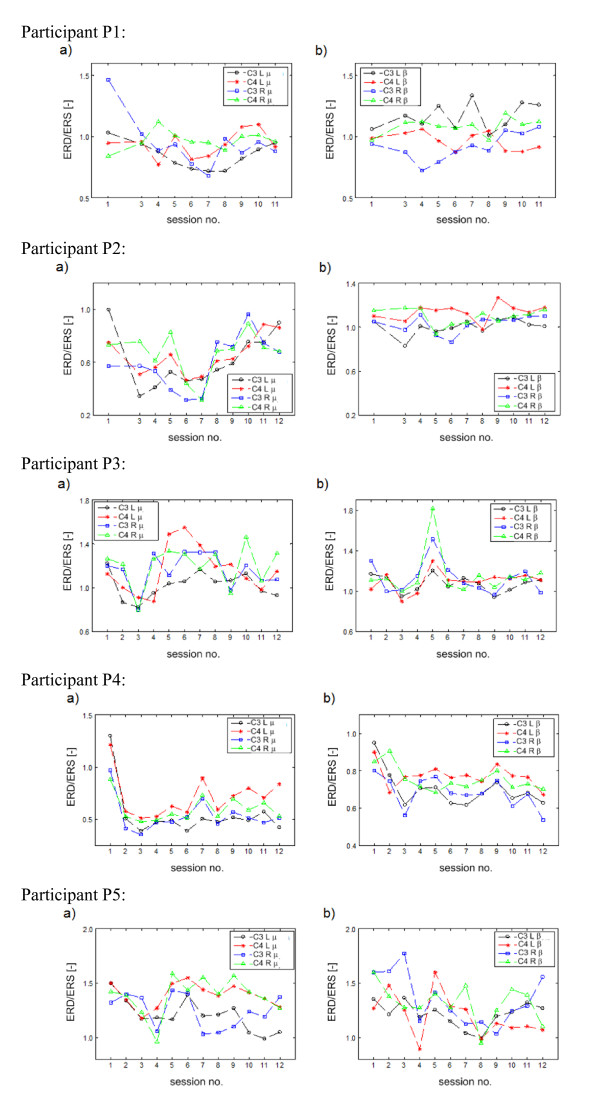
**Quantification of synchronized/desynchronized EEG activity within the adjusted μ and β bands over 12 recording sessions for all participants**: a) ${\text{ERD/ERS}}_{\text{μ}}^{\text{(C3L)}}$${\text{ERD/ERS}}_{\text{μ}}^{\text{(C4L)}}$, ${\text{ERD/ERS}}_{\text{μ}}^{\text{(C3R)}}$ and ${\text{ERD/ERS}}_{\text{μ}}^{\text{(C4R)}}$ b) ${\text{ERD/ERS}}_{\text{β}}^{\text{(C3L)}}$, ${\text{ERD/ERS}}_{\text{μ}}^{\text{(C4L)}}$, ${\text{ERD/ERS}}_{\text{β}}^{\text{(C3R)}}$, and ${\text{ERD/ERS}}_{\text{β}}^{\text{(C4R)}}$. The ratios in the μ band are represented as ${\text{ERD/ERS}}_{\text{μ}}^{\left(xy\right)}$ and that in the β band as ${\text{ERD/ERS}}_{\text{β}}^{\left(xy\right)}$, where *x *may denote the EEG channels C3 or C4 and *y *may denote either left upper limb MI (L) or right upper limb MI (R).

In order to analyse neurophysiological effects of BCI-supported MI practice, the ERD and ERS phenomena associated with MI were mainly targeted. The focus in the analysis of ERD/ERS phenomenon was on the quantification of the expected EEG desynchronization within the μ band (ERD_μ_) mainly on the contralateral side w.r.t. the MI task (i.e. in C3 for right MI trials and in C4 for left MI trials) and synchronization within the β band (ERS_β_) mainly on the ipsilateral side. In addition, the first non-feedback session and the last BCI session were compared using t-test at α = 0.05. The ERD/ERS ratios computed for all the participants are plotted in Figure 4. It is to be noted that the ratios in the μ band are represented as ${\text{ERD/ERS}}_{\text{μ}}^{\left(xy\right)}$ and that in the β band as ${\text{ERD/ERS}}_{\text{β}}^{\left(xy\right)}$, where *x *may denote the EEG channels C3 or C4 and *y *may denote either left upper limb MI (L) or right upper limb MI (R). The figure illustrates the ERD/ERS ratios in the tuned μ band in part (a), and the tuned β band in part (b) over all the EEG recording sessions for all five participants. The following inferences can be drawn from these plots.

• For P1, the significant drop in ${\text{ERD/ERS}}_{\text{μ}}^{\text{(C3R)}}$ and the enhancement of ${\text{ERD/ERS}}_{\text{β}}^{\text{(C3L)}}$ are the clearest observable trends for ERD/ERS ratios, especially when the first non-feedback and the last BCI session are compared.

• For P2, there is no conclusive evidence of a statistically significant difference between the first and the last session. However, the desynchronization within the μ band was a dominant phenomenon throughout all sessions.

• For P3, ERD/ERS did not show any significant changes between the first and the last session. There was a remarkable increase in both ${\text{ERD/ERS}}_{\text{β}}^{\text{(C3R)}}$ and ${\text{ERD/ERS}}_{\text{β}}^{\text{(C4R)}}$ in the session 5 only. Interestingly, this effect was not associated with any noticeable changes in the CA for right MI trials.

• For P4, except for the first non-feedback session, there was clear ERD within the μ band on both contralateral and ipsilateral channels during left and right MI trials. Rather unusually, desynchronization was also prevalent within the β band. For all the quantifiers, a significant drop from session 1 to session 12 was observed (i.e. deeper ERD state of μ and β rhythms).

• Finally, the ERD/ERS profiles for P5 demonstrated high variability and no significant differences between the first and the last session. It appears that both μ and β rhythms obtained from contralateral and ipsilateral locations were synchronized (quantifiers above 1) for most of the MI undertaken by P5.

Thus, the inspection of Figure 4 suggests a high degree of subject specificity in the evolution of ERD/ERS correlates over the course of MI practice sessions.

Correlations between participants' ERD/ERS and neurofeedback performance were also examined to verify the appropriateness of the features selection and classification procedures. For each participant, Pearson's product-moment correlation coefficients between the ERD/ERS measures and the CA obtained for either left or right MI trials, were computed over all the sessions with feedback. The coefficients are listed in Table 3. It is often expected that in all participants, the occurrence and strength of certain combinations of the lateralized ERD/ERS patterns (e.g., contralateral ERD_μ _and ipsilateral ERS_β _observed in healthy subjects performing MI tasks), would be strongly correlated to the degree of recognition and thus discrimination of the two MI trial types [9]. The analysis conducted in this work however did not provide consistent evidence for such stereotypical correlations across all participants. More specifically, the contralateral ERD_μ _effect was found to correlate with the classification performance only for P1 and P2. In particular, large negative correlation (*r *= -0.72) between ${\text{ERD/ERS}}_{\text{μ}}^{\text{(C3R)}}$ and the CA for right MI trials (CA^(R)^) was found in the participant P1. Similar relationships were identified for the participant P2 with the exception that the correlation involving ${\text{ERD/ERS}}_{\text{μ}}^{\text{(C3R)}}$ was lower (*r *= -0.58). For the left MI trials in P2, however, the contralateral ${\text{ERD/ERS}}_{\text{μ}}^{\text{(C4L)}}$ was positively correlated with the CA^(L)^. Other non-stereotypical correlations of the ERD/ERS_μ_effects with CAs included negative correlation (*r *= -0.61) between ${\text{ERD/ERS}}_{\text{μ}}^{\text{(C4R)}}$ and CA^(R) ^in P1, negative correlation between ${\text{ERD/ERS}}_{\text{μ}}^{\text{(C3L)}}$ and CA^(L) ^(*r *= -0.68) indicating ipsilateral EEG desynchronization within the μ band in P4, and positive correlation (*r *= 0.66) between ${\text{ERD/ERS}}_{\text{μ}}^{\text{(C4L)}}$ and CA^(L) ^in P5. The latter case suggests that the contralateral synchronization of the μ rhythm, and not the desynchronization as in conventional cases reported for healthy subjects [9], carried discriminatory features for recognizing left MI trials in P5. As for the MI-driven modulation of the EEG power within the β band, the correlations with the CA results also demonstrated a range of subject-specific patterns. The ipsilateral ERD/ERS_β _phenomena was found to consistently contribute to the classification of the respective MI trials only in P5. The results were then scrutinized in the context of stroke-related lateralized impairments that the subjects suffered. However, no consistent trends for this subject population were identified in this regard.

**Table 3:** Pearson's product-moment correlation coefficients for different possible pairings between a left/right CA and a μ/β band ERD/ERS ratio. Symbol (*) marks significant results (p <0.05)

		P1	P2	P3	P4	P5
Left MI	CA^(L)^vs${\text{ERD/ERS}}_{\text{μ}}^{\text{(C3L)}}$	0.21	0.81*	-0.17	-0.68*	0.18
	
	CA^(L)^vs${\text{ERD/ERS}}_{\text{μ}}^{\text{(C4L)}}$	0.27	0.88*	-0.12	0.03	0.66*
	
	CA^(L)^vs${\text{ERD/ERS}}_{\text{β}}^{\text{(C3L)}}$	-0.38	0.22	0.22	-0.46	0.05
	
	CA^(L)^vs${\text{ERD/ERS}}_{\text{μ}}^{\text{(C4L)}}$	-0.43	0.14	0.30	-0.25	-0.33

Right MI	CA^(R)^vs${\text{ERD/ERS}}_{\text{μ}}^{\text{(C3R)}}$	-0.72*	-0.58*	-0.09	-0.25	0.19
	
	CA^(R)^vs${\text{ERD/ERS}}_{\text{μ}}^{\text{(C4R)}}$	-0.61*	0.03	-0.49*	0.05	-0.50
	
	CA^(R)^vs${\text{ERD/ERS}}_{\text{β}}^{\text{(C3R)}}$	-0.36	-0.61*	0.18	0.26*	0.48*
	
	CA^(R)^vs${\text{ERD/ERS}}_{\text{β}}^{\text{(C4R)}}$	-0.22	-0.52	-0.13	-0.12	0.51*

In general, the analysis of the correlations between the strength of the MI induced lateralized ERD/ERS and the BCI CA performance demonstrated subject specific patterns. The ERD/ERS plots (cf. Figure 4) most certainly demonstrated that the MI practices resulted in asymmetric electrophysiological responses in frequency bands associated with μ and β rhythms [33]. This suggests that other discriminative ERD/ERS features in addition to the conventional ones linked to contralateral ERD_μ _and ipsilateral ERS_β _should also be included in the design of feature selection and classification procedures. It is therefore argued that the application of the proposed computational intelligence-based framework, implemented here with the use of type-2 fuzzy system, capable of effective learning from data (consisting of both contralateral and ipsilateral ERD/ERS features from μ and β bands) to maximize the classification performance, is a suitable approach in the context of the objectives of post-stroke MI practice.

### Rehabilitation Outcomes

**Figure 5:**
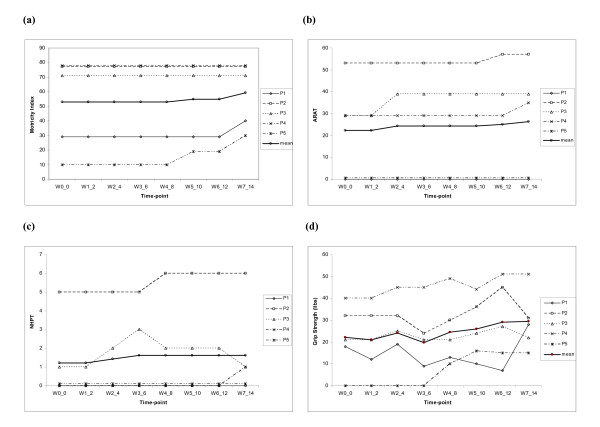
**Recording of rehabilitation outcome measures with respect to time-points *wi_j*, where *i *represents the week and *j *represents the session number**: **(a) **Motricity Index score (/100). (b) ARAT Score (/57). **(c) **NHPT Score (/6). **(d) **Grip strength (lbs.).

As seen in Figure 5a, two participants (P1 and P5), both with low initial scores at baseline, showed good improvement in McI scores. The others showed no change, but had greater scores at baseline, suggesting that there may have been a ceiling effect towards the higher end of the scale (Figure 5a). Across all the participants, there was a mean change of 6.2 (11.7%) with respect to the mean score (53) recorded at baseline in the week before the intervention began.

Out of the three participants (P2, P3 and P4) able to complete the ARAT test (Figure 5b), all demonstrated improvements in score, with two (P3 and P4) exceeding the MICD of 5.7 points. Across all the participants, there was a mean change of 4.0 (18.0%) with respect to the mean score (22.3) recorded at baseline in the week before the intervention began. The mean change was thus closely approaching the ARAT MICD score. In a similar study without BCI support reported in [3], where 32 chronic stroke sufferers participated in a controlled trial over 12 therapy sessions involving both PP and MI practice, there was a mean ARAT score improvement of 7.8 (SD = 5.1) on the baseline mean score of 18. In the current study, P2, P3 and P4 had ARAT score improvements of 4.0, 10.0 and 6.0 respectively and thus the improvements are in the similar range as reported in [3].

Only two participants were able to complete the NHPT test at all time-points (P2, P3, Figure 5c). The participant P3 was able to do so within the 120 second time period and performed the test consistently throughout the intervention period but then returned to baseline at the follow-up. The participant P4 could complete some NHPT tasks only in the follow-up session. Across all the participants, there was a mean change of 0.4 (33.3%) with respect to the mean score (1.6) recorded at baseline in the week before the intervention began..

All the five participants showed improvement in dynamometer grip strength (GS) at some time-point during the intervention period (Figure 5d). However, two participants (P2 and P3) showed a loss of grip strength towards the end of the intervention and returned closer to base-line by the follow-up session. The reasons for this finding are uncertain. Across all the participants, there was a mean change of 4.4 (20.0%) with respect to the mean score (22.2) recorded at baseline in the week before the intervention began..

In order to select a minimum number of outcome measures so that incremental recovery could be monitored across all participants, a Pearson's product-moment correlation coefficient (*r*) was computed for every possible pairing between left/right upper limb MI induced ERD/ERS ratio in μ/β band and a rehabilitation outcome measure recorded over the whole intervention period. Five sets of correlation coefficients are tabulated in the columns of Table 4 corresponding to five participants. The table includes only those rows of coefficients, in which at least one coefficient has a value equal to or more than 0.5, i.e. there is a large correlation between at least one participant's ERD/ERS ratio and an outcome measure score. As seen in Table 4 an associated ERD/ERS ratio had large correlation with GS (*r *= 0.77) and McI (*r *= 0.61) for P1; ARAT (*r *= 0.50) and NHPT (*r *= 0.50) for P2; ARAT (*r *= -0.61) and GS (*r *= -0.74) for P3; ARAT (*r *= -0.69) for P4; and McI (*r *= -0.76) and GS (*r *= -0.63) for P5. Since a ceiling effect was observed in McI outcomes for some participants, ARAT and GS (with underlined entries in Table 4) will be the best choice for monitoring of incremental recovery across all the five participants. It is also to be noted that the two participants, P2 and P3, who showed a loss of GS towards the end of the intervention returning closer to baseline, demonstrated consistent improvement on ARAT. However, there is a need to establish an MCID for GS.

**Table 4:** Pearson's product-moment correlation coefficients for different pairings between left/right upper limb MI ERD/ERS ratio in μ/β band and a rehabilitation outcome measure recorded over whole of the intervention period.

	P1	P2	P3	P4	P5
${\text{ERD/ERS}}_{\text{μ}}^{\text{(C3L)}}$ vs McI	0.31				**-0.52**

${\text{ERD/ERS}}_{\text{μ}}^{\text{(C3L)}}$ vs ARAT		0.45	**-0.61**	-0.22	

${\text{ERD/ERS}}_{\text{μ}}^{\text{(C3L)}}$ vs GS	0.12	-0.06	**-0.61**	0.05	**-0.50**

${\text{ERD/ERS}}_{\text{μ}}^{\text{(C4L)}}$ vs ARAT		**0.50**	0.12	-0.05	

${\text{ERD/ERS}}_{\text{μ}}^{\text{(C4L)}}$ vs NHPT		**0.50**	0.46	0.02	0.00

${\text{ERD/ERS}}_{\text{μ}}^{\text{(C4L)}}$ vs GS	-0.17	0.32	**-0.74**	-0.03	-0.05

${\text{ERD/ERS}}_{\text{μ}}^{\text{(C3R)}}$ vs GS	0.15	0.14	**-0.64**	-0.10	**-0.58**

${\text{ERD/ERS}}_{\text{μ}}^{\text{(C4R)}}$ vs GS	-0.27	-0.37	**-0.72**	0.20	0.02

${\text{ERD/ERS}}_{\text{β}}^{\text{(C3L)}}$ vs GS	-0.15	0.04	**-0.63**	0.01	-0.38

${\text{ERD/ERS}}_{\text{μ}}^{\text{(C4L)}}$ vs ARAT		0.23	0.30	**-0.69**	

${\text{ERD/ERS}}_{\text{μ}}^{\text{(C4L)}}$ vs NHPT		0.23	**0.53**	-0.48	0.00

${\text{ERD/ERS}}_{\text{μ}}^{\text{(C4L)}}$ vs GS	**0.77**	0.10	-0.21	-0.05	-0.38

${\text{ERD/ERS}}_{\text{β}}^{\text{(C3R)}}$ vs McI	**0.61**				-0.09

${\text{ERD/ERS}}_{\text{β}}^{\text{(C3R)}}$ vs ARAT		0.46	-0.33	**-0.50**	

${\text{ERD/ERS}}_{\text{β}}^{\text{(C3R)}}$ vs GS	-0.42	0.40	-0.35	-0.08	**-0.63**

${\text{ERD/ERS}}_{\text{β}}^{\text{(C4R)}}$ vs McI	0.17				**-0.76**

${\text{ERD/ERS}}_{\text{β}}^{\text{(C4R)}}$ vs NHPT		0.36	**0.60**	-0.23	0.00

${\text{ERD/ERS}}_{\text{β}}^{\text{(C4R)}}$ vs GS	-0.10	0.05	-0.38	0.26	**-0.51**

Some entries are blank because no coefficient could be computed, as the corresponding outcome scores remained unchanged.

### Visual Aanlog Scores for Fatigue and Mood

**Figure 6:**
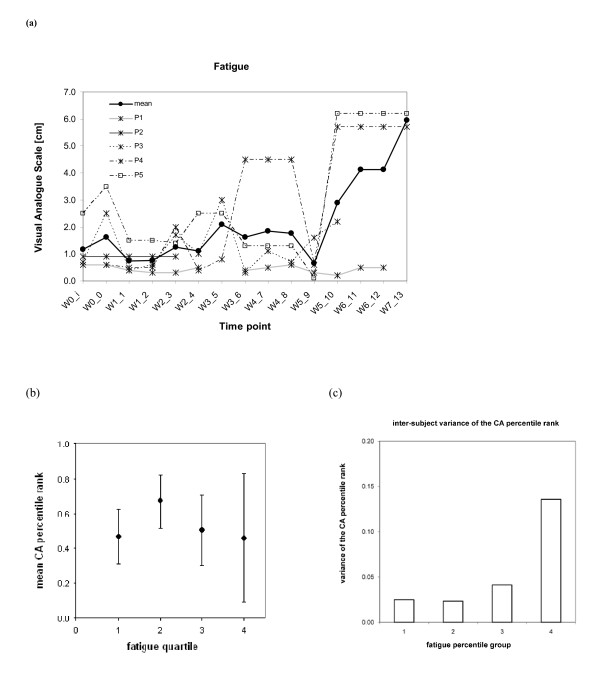
**Monitoring of Fatigue**: **(a) **Visual analog scores **(**VAS) for fatigue plotted with respect to time-points *wi_j*, where *i *represents the week and *j *the session number. **(b) **Dependency between CA results and fatigue VAS-plot of the subject-wise CA percentile rank (inter-subject mean with standard deviation) matched with fatigue VAS quartiles (i.e. inter-quartile ranges). **(c) **Dependency between CA results and fatigue VAS-plot of the inter-subject variance of subject-wise CA percentile ranks matched with fatigue VAS quartiles (i.e. inter-quartile ranges).

There were moderate increases in the fatigue (Figure 6) reported by three of the participants. This resulted in a group mean change of +4.77 cm. Although it is possible that the increase was caused by factors external to the therapy, it could also be due to the exercises undertaken over two hour long sessions (including time required in preparation). However, there are substantial fluctuations in fatigue over the treatment period. Since no reassessments or further follow up recordings were made, the longer term effects of the intervention fatigue are uncertain. In order to examine potential dependencies between the CA results and the fatigue scores reported in the study, an attempt to correlate these quantities was made. Due to varying ranges of CAs in different subjects, individual percentile ranks (0-1) were determined, which provided more intuitive measure of the performance level for every subject. These values were then matched with fatigue scores grouped in four inter-quartile ranges (independent division for each subject into four bins - below the first quartile, from the first to the second quartile, from the second to the third quartile and above the third quartile). Finally, the CA percentile ranks were averaged within each range of fatigue quartile and subsequently, the mean and the standard deviation for all subjects were evaluated. This is depicted in the Figure 6b. As can be noticed, there do not appear any clear interaction terms. The only observable trends are for the last three inter-quartile ranges of fatigue, where growing VAS score levels correspond to a decrease in the CA ranks for fatigue. This interpretation has been further reinforced in Figure 6c where a plot is drawn between the inter-subject variance of subject-wise CA percentile ranks and VAS fatigue score quartiles. Based on this plot, it can be argued that higher level of fatigue can contribute to a larger variability in the BCI performance among the subjects. It may also be argued that growing fatigue has increasingly varying effect on different subjects. However, the observations can only be treated as a trend without convincing statistical evidence.

**Figure 7:**
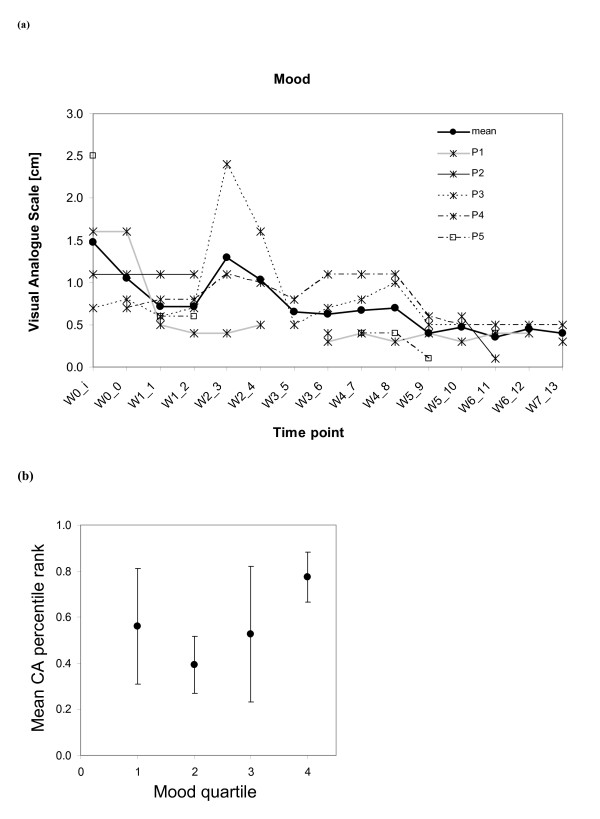
**Monitoring of Mood**: **(a) **Visual analog scores (VAS) for mood plotted with respect to time-points *wi_j*, where *i *represents the week and *j *the session number. (b) Dependency between CA results and mood VAS-plot of the subject-wise CA percentile rank (inter-subject mean with standard deviation) matched with mood VAS quartiles (i.e. inter-quartile ranges).

As far as mood changes are concerned, all of the participants showed improvement in mood (Figure 7) during the intervention period with a group mean change close to -0.8 cm. This change can be considered as clinically significant. Similar to the fatigue above, the CA percentile ranks are plotted in Figure 7b for mood. As expected, main observable trends are for the last three inter-quartile ranges of mood, where growing VAS score levels correspond to an increase in the CA ranks for mood.

### Qualitative comments

The participants were overall pleased to have taken part in the study despite its feasibility evaluation aspect. Most of them found it beneficial in terms of the enhanced concentration and one individual reported an improvement of certain motor functions in his affected hand (subjective perception). Two subjects found the treatment sessions excessively lengthy and tiring, particularly if they were held in the afternoon. All of the subjects expressed willingness to participate in potential follow-up studies. With regard to the use of BCI technology, subjects did not experience any significant difficulties in embracing the neurofeedback paradigm. However, most of them suggested the need for more interesting, challenging and thus more immersive computer game scenarios.

## Discussion and Conclusions

With the help of a pilot trial, the paper has presented a feasibility study of an EEG-based BCI generated neurofeedback to support patient engagement during an MI practice performed as part of a post-stroke rehabilitation protocol combining both PP and MI practice of rehabilitation tasks. The protocol used a BCI controlled ball-basket game based neurofeedback for confirming the patient engagement on-line. Five individuals suffering from stroke for more than a year participated in the pilot trial involving up to twelve treatment sessions. The on-line CA of MI induced SMR patterns in the form of ERD and ERS, for novice participants was in a moderate of range 60-75% within the limited 12 half an hour long BCI training sessions undertaken as part of treatment sessions. A detailed analysis of EEG data demonstrated that two different types of MI practices resulted in hemispherically asymmetric electrophysiological responses in frequency bands corresponding to μ and β rhythms, which clearly demonstrated that both hemispheres were stimulated in all participants. There also existed a high correlation between the CA rates and the ERD/ERS ratios demonstrating that the hemispheric asymmetry in both μ and β bands contributed to BCI CA rates. However, for only two participants, the ERD change was statistically significant between the first session and the last session.

The study found improvements in some of the functional outcome measure scores for all the participants as a result of undergoing the rehabilitation protocol. The improvements in ARAT for two of the participants exceeded the MCID limit, while its mean change was nearly approaching the MCID limit. Based on the Pearson's correlation coefficient computation for every possible pairing between left/right upper limb MI induced ERD/ERS ratio in μ/β band and a rehabilitation outcome measure score, it was found that the scores of two outcome measures, ARAT and GS, have large correlation with ERD/ERS ratios of all the participants and these two will be sufficient to monitor incremental functional gains during the intervention. However, an MCID needs to be established for GS. As expected, most participants were suffering from fatigue. As far as interaction of the fatigue scores with the CAs is concerned, it can be argued that higher level of fatigue can contribute to a larger variability in the BCI performance among the subjects. Nevertheless, there was significant improvement in average mood over the treatment sessions. Participants in general appeared very enthusiastic about participating in the study and regularly attended all the sessions. There was no drop out at all.

The origins of rather moderate CA values reported in the experiments are multifarious-subjects were novice BCI users, they could have difficulties maintaining high concentration and performing consistent MI throughout the entire experimental session, or the lateralization of the MI related EEG correlates that the BCI relies on could be affected due to post-stroke brain lesion. It maybe possible to improve the CA performance by adapting the BCI system to address specificities of MI induced EEG patterns recorded from stroke rehabilitants. For significant enhancement in CA rates, the study should run for much larger number of sessions, i.e. at least 20 or more sessions. Overall, however, the crucial observation is the fact that the moderate BCI classification performance did not impede the positive rehabilitation trends as quantified with the rehabilitation outcome measures adopted in this study. Therefore it can be concluded that the BCI supported MI practice is a feasible intervention as part of a post-stroke rehabilitation protocol combining both PP and MI practice of rehabilitation tasks. It is however yet to be ascertained whether the enhanced rehabilitation gain is primarily because of BCI neurofeedback, as the positive impact of MI practice without feedback has been reported in a recent study [3]. Additionally it is to be noted that the scope of the final conclusions is limited by the small sample size and the lack of a control group. To address these issues, it is proposed to perform a more extensive follow-up study in near future.

## Competing interests

The authors declare that they have no competing interests.

## Authors' contributions

GP conceived the initial idea, obtained ethical approval and led the pilot study. PH undertook algorithmic developments, data analysis and performed most of BCI related experimental tasks with some help from GP and DC. PH, JC, and SM supported the content and delivery of the MP intervention; also provided advice and training on the outcome measures used. GP wrote the first draft and all authors revised and approved the final manuscript.
